# Species Differentiation on a Dynamic Landscape: Shifts in Metapopulation Genetic Structure Using the Chronology of the Hawaiian Archipelago

**DOI:** 10.1007/s11692-012-9184-5

**Published:** 2012-05-15

**Authors:** George K. Roderick, Peter J. P. Croucher, Amy G. Vandergast, Rosemary G. Gillespie

**Affiliations:** 1 Department of Environmental Science, Policy, and Management, University of California, 130 Mulford Hall, Berkeley, CA 94720-3114 USA; 2 U.S. Geological Survey, Western Ecological Research Center, San Diego Field Station, 4165 Spruance Road, Suite 200, San Diego, CA 92101 USA

**Keywords:** Speciation, Shifting mosaic, Metapopulations, Founder events, Genetic revolutions, Adaptive radiation, Dispersal

## Abstract

Species formation during adaptive radiation often occurs in the context of a changing environment. The establishment and arrangement of populations, in space and time, sets up ecological and genetic processes that dictate the rate and pattern of differentiation. Here, we focus on how a dynamic habitat can affect genetic structure, and ultimately, differentiation among populations. We make use of the chronology and geographical history provided by the Hawaiian archipelago to examine the initial stages of population establishment and genetic divergence. We use data from a set of 6 spider lineages that differ in habitat affinities, some preferring low elevation habitats with a longer history of connection, others being more specialized for high elevation and/or wet forest, some with more general habitat affinities. We show that habitat preferences associated with lineages are important in ecological and genetic structuring. Lineages that have more restricted habitat preferences are subject to repeated episodes of isolation and fragmentation as a result of lava flows and vegetation succession. The initial dynamic set up by the landscape translates over time into discrete lineages. Further work is needed to understand how genetic changes interact with a changing set of ecological interactions amongst a shifting mosaic of landscapes to achieve species formation.

## Introduction

The structuring of populations is critical to an understanding of the process of speciation. While evolutionary studies typically focus on longer time scales, the rapidity of adaptation has highlighted the importance of integrating ecological and evolutionary approaches to understand the initial stages of differentiation (Schoener 2011). The establishment of small populations, and fragmentation of larger ones, is arguably the most important aspect in the initiation of species divergence and adaptive radiation, although research has progressed along two largely independent avenues—genetic and ecological. The former builds on ideas of founder effects (Carson and Templeton 1984) and metapopulation dynamics (Gavrilets et al. 2000), the latter on species-area relationships (Losos and Schluter 2000) and food web theory (Schoener and Spiller 2010). Here, we show how the unique chronological arrangement of habitats on the youngest islands of the Hawaiian archipelago allows insights into the early stages of the diversification process. We focus on population genetic aspects of diversification, but incorporate discussion of the ecological context.

Migration, or gene flow, and population size are important factors dictating population establishment and persistence. Gene flow allows populations to become established, and may promote evolution by spreading new genes and combinations of genes throughout a species’ range; alternatively, gene flow can constrain evolution by preventing adaptation to local conditions (Slatkin 1987). The importance of migration, or lack thereof, in differentiation leading to speciation, in turn depends on the structure and dynamics of available habitats (Carson et al. 1990). In ephemeral habitats, migration is necessary for populations to persist (see Denno et al. 1991), while in more stable habitats, not only is gene flow unnecessary, but limited gene flow can allow populations to diverge by chance alone (Wright 1968). The island system we examine here allows assessment of the role of habitat dynamics in both space and time in dictating genetic population structure and differentiation.

### Can Founder Events Alone Trigger Adaptive Radiation?

The genetics of speciation has a long history associated with founder events (Provine 2004). During a founder event, random genetic drift might be expected to result in a massive loss of genetic variability. So, how do newly founded populations overcome low genetic diversity and expected low evolutionary potential, typically associated with extinction risk, to become established outside of their native range (Roman and Darling 2007)? Several factors have been proposed that may mitigate some or all of the negative genetic impacts associated with founder events and the establishment of small populations, whether linked to current invasions or the initial founding of incipient species. First, provided there is rapid growth subsequent to the bottleneck, then relatively little genetic variability may be lost (Nei et al. 1975), with the new population containing sufficient genetic variation upon which natural selection can build novel character states (Carson 1990). Second, non-additive variance might be converted to additive genetic variance, resulting in a corresponding increase in response to selection for some traits (Neiman and Linksvayer 2006). The different genetic environment of the newly founded populations can then yield more variable evolutionary responses (Rundle 2003). It has been argued that, as a result of these effects, speciation can occur through a “genetic revolution” (Mayr 1954) or “founder flush” (Carson 1975), often associated with rapid growth of the new population. However, while verbal and theoretical arguments have been developed to show that speciation can occur through founder events alone, empirical evidence—both laboratory and field—for this process is lacking (Charlesworth and Smith 1982; Rundle 2003; Butlin et al. 2012). An additional genetic argument for a positive role of founder events in enhancing fitness of a set of populations is the idea of genetic purging: among outbreeding sexual species, rare deleterious alleles with lethal effects can be purged by selection in bottlenecked populations, potentially allowing recovery of fitness (Kristensen and Sorensen 2005). However, recent studies suggest that the effects of purging are unpredictable and rarely strong enough to eliminate inbreeding depression (Boakes et al. 2007). Despite a lack of empirical support for the role of bottlenecks in facilitating differentiation in natural populations (but see for laboratory populations, Regan et al. 2003; Nanda and Singh 2011) some theoretical evidence exists to suggest that bottlenecks may play a substantial role in the speciation process (Templeton 2008), though the genetic basis is still unclear and the ecological context is critical.

### A Role for Ecological Processes? Metapopulations and Admixture in a Dynamic Landscape

Carson et al. (1990) were the first to hypothesize that metapopulation structure may play a role in concert with founder effects to facilitate diversification. They suggested that the temporal cycles of fragmentation due to lava flows on the youngest island of the Hawaiian archipelago have played a key role in creating and maintaining genetic differences among populations and, ultimately, in the evolution of new character states and species (Carson and Sato 1969; Carson and Templeton 1984; Carson et al. 1990). While the importance of the geological environment in facilitating isolation is now more thoroughly documented (Thornton 1984; Gillespie and Croom 1995; Givnish et al. 1995; DeMeyer 1996; Vandergast et al. 2004; Goodman et al. 2012), the nature of the metapopulation dynamics in facilitating diversification is not obvious.

A clearer understanding of how aspects of early-stage colonization history may affect the genetic capacity for adaptive responses is required (Eales et al. 2010). Some insights into parameters dictating success of establishment following a founder event have emerged from studies of recent biological invasions. In particular, negative effects of genetic founder effects may be offset if different colonization events result in multiple genotypes within the introduced population. Such effects have been documented in a variety of organisms, including plants, lizards, and arthropods (Ellstrand and Schierenbeck 2000; Kolbe et al. 2004; Chen et al. 2006; Boubou et al. 2012), and highlight the potential role of admixture among successively introduced populations in providing the genetic variation to allow adaptive evolution (Suarez and Tsutsui 2008).

### Can Ecological Processes Lead to Species Proliferation in Dynamic Habitats?

Repeated cycles of habitat expansion and contraction may serve to allow species to alternately expand their ranges and then become isolated. In the metapopulation and admixture examples above, these processes act over ecological time, but how they might translate into new species is not clear. However, similar phenomena of shifts driven by glacial fluctuation in lakes (Weir and Schluter 2004) and climate change with elevation (Schoville et al. 2012) have been suggested as causing “species pumps”, in which species are presented with cycles of sympatry in glacial periods and allopatry in interglacial periods. Modeling studies also point to the importance of habitat dynamics on divergence in the context of migration and selection. For example, Vuilleumier et al. (2010) have shown by computer simulation that in fragmented habitats both migration type and pattern can have large impacts on the probability of fixation of alleles when demes differ in effective size or selection pressures. The Hawaiian Islands provides a model system in which one can observe the entire spectrum of ecological processes within a temporal and spatial context, allowing examination of the combined effects of isolation and genetic structure, and ultimately differentiation and species formation, in a dynamic landscape that has a well-defined chronology.

### Dynamic Landscape and Chronological Arrangement of the Hawaiian Islands

**Fig. 1:**
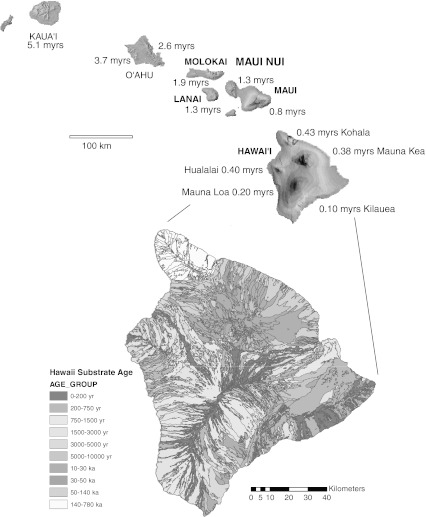
Map of Hawaii, or the “Big Island”, showing dissection from lava flows, much of it occurring over the last century on the slopes of Mauna Loa, the second youngest volcano on the island. Kohala is the oldest volcano (0.43 MY), then Hualalai (0.40 MY), Mauna Kea (0.38 MY), Mauna Loa (0.20 MY), and Kilauea (0.10 MY). The *inset* shows the entire archipelago. Each island of Maui Nui has a single volcano except for Maui, which has two; these volcanoes range in age from Molokai (1.9 MY), through Lanai and West Maui (1.3 MY) to East Maui (0.8 MY). After USGS Hawaiian Volcano Observatory maps, http://hvo.wr.usgs.gov/maunaloa/hazards/historicalflows.html and http://pubs.usgs.gov/gip/hazards/maps.html (Color figure online)

The Hawaiian Islands (Fig. 1) are topographically complex and extremely geographically isolated, characteristics which have allowed adaptive radiation in multiple groups over similar time periods (Wagner and Funk 1995; Roderick and Gillespie 1998). The islands are formed over a roughly stationary hot spot near the center of the Pacific plate, which moves northwestward creating a chronosequence from submerged seamounts >70 my in the northwest to new lava flows in the southeast (Carson and Clague 1995). Each island is a composite of several shield volcanoes that formed in geological sequence. As new layers cooled, they provided substrate for colonization by taxa, which were then subject to repeated events of local extirpation and recolonization as the volcanoes grew. This process can be observed today on the youngest island, the “Big Island” of Hawaii, where the still-flowing and highly fragmented landscape of Mauna Loa (Wolfe and Morris 1996) gives snapshots of the geographic conditions under which each island formed. The real-time observations of conditions on the Big Island offer a realistic picture of the fragmented conditions under which many of Hawaii’s classic adaptive radiations originated and an age-structured landscape to study community assembly. Here, we focus on the dynamic landscape of the Big Island in comparison with the next oldest volcano on Maui Nui.

The Big Island is the largest in the archipelago and the youngest (Fig. 1). On the youngest and most active of these volcanoes, Mauna Loa and Kilauea, lava flows bury surfaces at rates of about 40 and 90 % per 1,000 years respectively (Carson et al. 1990). Ongoing volcanic activity has created a shifting mosaic of habitats as large areas of forest are destroyed or fragmented into small habitat “islands” (called *kīpuka*) when lava flows over or around them. After a lava flow cools, the forest gradually regenerates, receiving founders from other intact areas. A mature, closed canopy forest has been estimated to develop in 300–3,000 years on new flows, with temporal differences depending on abiotic conditions (e.g., slope, aspect, altitude, and lava type) and biotic factors. Detailed work on dating of island ages, the soils and lava flows by the USGS (Trusdell et al. 1996; Price and Clague 2002) has provided an explicit temporal context for the islands, allowing the calibration of divergence time estimates between populations based on molecular data (Vandergast et al. 2004; Goodman et al. 2012).

The closest group of islands to the northwest, representing the next (older) level in the chronosequence, is Maui Nui, a composite of 4 separate volcanic islands, Maui, Molokai, Lanai, and Kahoolawe (Fig. 1). It was a single large landmass for more than 75 % of its history much like the Big Island is today (Price and Elliott-Fisk 2004). Glacially mediated fluctuations in sea level have alternately flooded and exposed the land connecting islands of the complex. Within Maui Nui, diversity and endemism patterns support treating the island complex as a single unit rather than individual islands (Price 2004).

### Hypothesized Effects of Dynamic Landscape Chronology on Species Differentiation

Here we use the model system provided by the Hawaiian Islands to conduct a preliminary test of predictions regarding the role of the dynamic landscape in fostering differentiation. We focus on Hawaiian spiders, which make excellent targets for examination of the entire process from widely distributed populations on the youngest volcanoes, to highly structured populations and incipient species on the next youngest island (Gillespie 2004). We have chosen a set of 6 endemic spider lineages that differ in habitat affinities to examine how the dynamic landscape has played a role (1) in retention of genetic diversity, thus serving as a crucible to maintain and promote genetic diversity; and (2) in isolation, and hence differentiation, of populations ultimately leading to speciation as volcanoes age (Carson et al. 1990). Two general hypotheses emerge.

1. *Active volcanism fosters genetic diversity*. Studies have shown that lava flows divide habitats, causing isolation, yet this isolation is transient and the open habitat subsequently becomes available for further colonization. Our hypothesis is that this dynamic creates the ideal setting for promoting and maintaining genetic diversity because it provides for isolation and local selection, but also an avenue for repeated colonization. Therefore, we predict (a) that population structure in the Big Island region of more recent volcanic activity should reflect significant dispersal that can foster genetic diversity across populations. Also, (b) populations of species that are habitat specialists in subdivided forest habitats should be more structured than populations of habitat generalists (Vandergast et al*.* 2004).
2. *Volcanic maturation promotes opportunity for isolation*. As the Hawaiian Islands move away from the hotspot, they subside (Carson and Clague 1995) and we hypothesize that this process leads to isolation by distance, and ultimately differentiation. The islands of Maui Nui can be used to examine the next stage in the chronology, and how genetic diversity may become subdivided over time. The topography of Maui Nui reached its maximum extent around 1.2 MY (Price and Elliott-Fisk 2004). However, unlike the current Big Island, Maui Nui had a much greater area below 1,000 m than Hawai’i. The saddles between the volcanoes of Maui Nui would likely have created a band of rainforest on the windward slopes and would have gradually become more dissected as the saddles subsided to their current positions near sea level. Therefore, while connections have been maintained between volcanoes by lowland forest, taxa that are restricted to higher elevation habitats are likely to have been isolated for much longer on Maui Nui. Therefore, we predict (a) that genetic differentiation should be greater among populations on volcanoes of Maui Nui than those of the Big Island; and (b) that species differentiation should be more pronounced in taxa that are restricted to forest habitats at middle to higher elevations (approx. 1,000–2,000 m). Those species occurring at lower elevations would not have been isolated by the subsidence of the volcanoes. Finally (c), the distance between the Maui Nui complex of volcanoes and the volcanoes of the Big Island should also contribute to isolation.

## Methods

### Study Sites and Organisms

This study included specimens collected in localities on the islands of Maui Nui and the Big Island in the Hawaiian Archipelago (Fig. 1). The localities encompass wet and dry forests habitats and are described in more detail in Table 1. The 6 spider lineages studied include 4 habitat specialists and 2 habitat generalists:

**Table 1:** Volcanoes, collection localities, sample size, and available data for the 6 spider lineages included in this study

Island	Volcano (associated localities)	Latitude, longitude (approx.)	Elev. (approx.)	Habitat description	Allozyme data: lineage, species (n, loci)	mtDNA data: lineage, species (n, basepairs)
Maui Nui	Molokai (Kamakou)	21 06N, 156 54W	1,200 m	Montane wet	L1 *Th. grallator* (8, 8) L5 *T. quasimodo* (8, 9)	L1 *Th. grallator* (4, 1,270)
	Lanai	20 49N, 156 53W	1,030 m	Montane mesic & wet		L4 *T. macracantha* (1, 605)
	West Maui	20 56N, 156 37W		Montane wet	L1 *Th. grallator* (16, 8)	L1 *Th. grallator* (8, 1,270)
	Haleakela (Kipahulu)	20 41N, 156 5W	1,200 m	Montane wet		L4 *T. macracantha* (3, 605) L6 *T. hawaiiensis* (1, 528)
	Haleakela (Lower Waikamoi)	20 48N, 156 14W	1,340 m	Montane mesic	L1 *Th. grallator* (96, 8) L3 *T. kikokiko* (9, 9)	L1 *Th. grallator* (66, 1,270) L2 *A. alepeleke* (2, 420) L3 *T. kikokiko* (2, 607) L4 *T. brevignatha* (2, 605)
	Haleakela (Upper Waikamoi)	20 46N, 156 13W	1,900 m	Montane wet	L5 *T. quasimodo* (19, 9)	L2 *A. corniger* (2, 420) L2 *A. laau* (4, 420) L5 *T. quasimodo* (2, 439)
	Haleakela (Auwahi)	20 38N, 156 20W		Montane dry & mesic		L2 *A. corniger* (3, 420) L3 *T. kikokiko* (2, 607)
Big Island	Kohala	20 05N, 155 45W	1,150 m	Montane wet	L1 *Th. grallator* (32, 8)	L1 *Th. grallator* (17, 1,270) L4 *T. brevignatha* (2, 605) L5 *T. quasimodo* (5, 439)
	Hualalai	19 41N, 155 52W	1,100 m	Montane mesic & wet	L5 *T. quasimodo* (2, 9)	L4 *T. brevignatha* (1, 605) L5 *T. quasimodo* (6, 439)
	Mauna Kea (Laupahoehoe)	19 56N, 155 16W	1,260 m	Montane wet		L4 *T. brevignatha* (1, 605) L5 *T. quasimodo* (14, 439)
	Mauna Loa (Honomolino)	19 11N, 155 51W	1,500 m	Montane mesic		L4 *T. brevignatha* (6, 605) L5 *T. quasimodo* (1, 439)
	Mauna Loa (*kīpuka* on the Saddle Road)	19 40N, 155 20W	1,550 m	Montane wet	L1 *Th. grallator* (50, 8) L3 *T. anuenue* (12, 9) L5 *T. quasimodo* (9, 9)	L1 *Th. grallator* (63, 1,270) L2 *A. waikula* (2, 420) L3 *T. anuenue* (162, 607), 9 pops L4 *T. brevignatha* (43, 605), 5 pops L5 *T. quasimodo* (111, 439), 8 pops L6 *T. hawaiiensis* (1, 528)
	Mauna Loa (Kahaualea)	19 25N, 155 08W	530 m	Lowland wet		L6 *T. hawaiiensis* (1, 528)
	Kilauea (Thurston)	19 24N, 155 14W	1,220 m	Montane wet	L1 *Th. grallator* (30, 8)	L1 *Th. grallator* (55, 1,270) L2 *A. waikula* (3, 420) L2 *A. hiwa* (1, 420) L6 *T. hawaiiensis* (1, 528)
	Kilauea (Puu Makaala)	19 24N, 155 14W	1,220 m	Montane wet	L5 *T. quasimodo* (16, 9)	L2 *A. waikula* (1, 420) L2 *A, hiwa* (1, 420) L3 *T. anuenue* (6, 607) L4 *T. brevignatha* (1, 605) L5 *T. quasimodo* (10, 439)

#### Habitat Specialists

L1, *Theridion grallator* (the Hawaiian happy face spider) on Maui Nui and the Big Island. *T. grallator* is a resident of the wet and mesic forests on all high islands of the archipelago except Kauai and has a web that consists of a scanty, two-dimensional layer of silk covering the entire underside of a leaf (Gillespie and Tabashnik 1994).

L2, *Ariamnes hiwa* and *A. waikula* on the Big Island and their sister species *A. corniger, A. alepeleke* and *A.* *laau* on Maui. The dark-colored *A. hiwa* and gold *A. waikula* are both endemic to the Big Island where they occur in wet forest habitats at middle elevations. *Ariamnes hiwa* occurs low down near the ground often inside rocky crevices, and *A. waikula* is found under larger leaves in the forest. Among the 3 species endemic to Maui, *A. laau* occurs in wet forest habitats at middle and high elevations, *A. alepeleke* in wet forest at middle elevations, and *A. corniger* in both wet and dry forest sites at middle and high elevations (Gillespie and Rivera 2007).

L3, *Tetragnatha anuenue* on the Big Island and its sister *T. kikokiko* on Maui. Neither species builds webs, and both are endemic to their respective islands. *Tetragnatha kikokiko* is a “small brown” species (Gillespie 2004), and occurs in dry and mesic forest at middle elevations while *T. anuenue* is a strikingly color polymorphic species found in middle elevation wet forest (Gillespie 2002).

L4, *Tetragnatha brevignatha* on the Big Island and Maui, and its sister species *T. macracantha* on Maui and Lanai. Neither species builds a web, both being representatives of the “green” ecomorph in the “spiny leg” clade (Gillespie 2004); they occur in wet and mesic forests, where they are generally found under leaves (Gillespie 1991).

#### Habitat Generalists

L5, *Tetragnatha quasimodo* on the Big Island and Maui Nui. *Tetragnatha quasimodo* does not build a web. It is a habitat generalist, which occurs in wet and dry forests from 700 to 2,300 m elevation with a distribution across all the islands except Kauai (Gillespie 1991).

**Fig. 2:**
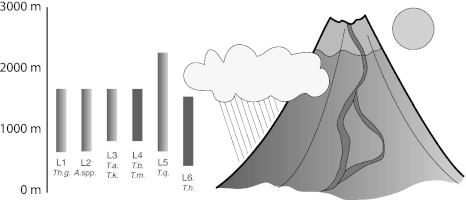
Habitat affinities and elevational distributions for 6 spider lineages included in this study. Graphic shows wet (*dark green*) and dry (*yellow*) habitats created by prevailing winds and rain shadows and how lava flows form habitat patches, or *kīpuka*. Spider lineages sampled include 4 habitat specialists (see text): L1 *Theridion grallator* on multiple islands, L2 *Ariamnes hiwa* and *A. waikula* on the Big Island and their sisters *A. corniger, A. alepeleke* and *A. laau* on Maui, L3 *Tetragnatha anuenue* on the Big Island and its sister *T. kikokiko* on Maui, L4 *T. brevignatha* on the Big Island and Maui, and its sister species *T. macracantha* on Maui and Lanai; and 2 habitat generalists found on multiple islands: L5 *T. quasimodo*, which occurs on wet and dry forests and L6 *T. hawaiensis* which inhabits low elevation forests (Color figure online)

L6, *Tetragnatha hawaiensis* on the Big Island and Maui Nui (Table 1; Fig. 2). *Tetragnatha* *hawaiensis* is a web spinner, and another habitat generalist, inhabiting low elevation forests on all high islands across the archipelago (Gillespie et al. 1994).

### Molecular Markers

#### *Allozyme data*

Allozymes were scored for *Tetragnatha* species for 9 loci and *Theridion grallator* for 8 loci, following procedures outlined in Gillespie and Oxford (1998). Allozyme data for *Theridion grallator* are provided in Gillespie and Oxford (1998) and for *Tetragnatha* in Gillespie (2004).

#### *Mitochondrial COI*

Sequences of mitochondrial DNA cytochrome oxidase I were obtained for all species following procedures in Gillespie (2004) and Croucher et al. (2012). For *T. grallator*, mtDNA sequences were a concatenated set of COI, 16S and ND1 (Croucher et al. 2012). Sequence lengths examined in each species are listed in Table 1.

### Geological History and Genetic Population Structure

To test the hypotheses (1) that *active volcanism fosters genetic diversity* and (2) that *volcanic maturation promotes opportunity for isolation* we estimated standardized genetic variance, $\Upphi_{ST}^{'}$, and migration rates, *N*_*e*_ *m*, among populations. Calculations are described below. If *active volcanism fosters genetic diversity*, we predict (a) that in the *kīpuka* region characterized by a metapopulation structure of population extinction and recolonization, genetic variance among populations should be low and migration high. Because only the Big Island is active volcanically, there is no ideal geographic comparison of the same scale elsewhere in the Hawaiian Islands; a previous study compared populations in the forest patches with neighboring continuous forest (Vandergast et al. 2004). We also predict (b) that population subdivision caused by volcanic activity should be more pronounced among specialists of forested habitats (L1, L3, L4), than among populations of the habitat generalist (L6, *T. quasimodo*) found in the *kīpuka* region.

If *volcanic maturation promotes opportunity for isolation*, we predict (a) more genetic variance and less migration among populations on the older Maui Nui than on the younger Big Island. Further, because the lower elevation habitats of Maui Nui have been continuous for longer than the higher elevation habitats, we predict more subdivision in mid-high elevation species (L3, L4) than species that also inhabit lower elevations (L1, L2, L5). We also predict increasing isolation by distance with volcanic maturation, from populations near active volcanism on the Big Island, to between volcanoes on the Big Island and Maui Nui, to between islands.

#### Standardized Genetic Variance, $\Upphi_{ST}^{'}$

To make comparisons across species, standardized estimates of genetic variance among populations were obtained following the approach of Miermans (2006). Using the AMOVA procedure performed in Arlequin (Schneider et al. 2000), we first computed the genetic variance among populations, $\Upphi_{ST}$. The data were recoded so that no alleles were shared among populations (but within population variation remains the same) and the AMOVAs were rerun to get an estimate of $\Upphi_{ST(\max )}$—the maximum possible value of $\Upphi_{ST}$. This value was used as a divisor to yield a standardized estimate of $\Upphi_{ST}$: $\Upphi_{ST}^{'}$. For mtDNA, only the polymorphic sites were used in the AMOVA, which were extracted through data manipulation in R (R Development Core Team 2011) using ape (Paradis et al. 2004) and adegenet (Jombart 2008). The statistical significance of $\Upphi_{ST}$ > 0 was tested using 10,000 permutations. It is not technically possible to directly compute a *p* value for the $\Upphi_{ST}^{'}$ estimate (Meirmans 2006).

#### Migration Rates, *N*_*e*_*m*

For species collected in at least 2 localities (Table 1), pair-wise migration rates among populations were estimated using a Bayesian coalescent framework as implemented in Migrate-N version 3.2.7 (Beerli and Felsenstein 1999, 2001; Beerli 2005). The program was used to estimate migration rates between pairs of populations (*N*_*e*_ *m*), where *N*_*e*_ is the effective population size, *m* is the migration rate between two populations. For each group of populations examined, a full migration model was examined, allowing unrestricted migration among all populations. Migrate-N runs for both the mtDNA and allozyme data consisted of a burn-in discarding 50,000 genealogies per chain. Five replicates were run, each sampling every 200 steps and recording 20,000 genealogies for the mtDNA data and 10,000 genealogies for the allozyme data such that 20,000,000 (mtDNA) and 10,000,000 (allozymes) genealogies were visited. An adaptive heating scheme, spread across four MCMC chains was employed and the mutation rate among allozyme loci was allowed to vary (as estimated from the data by the software). For each species at a given geographical scale the range of the median posterior values of *N*_*e*_ *m* among all pairs of populations is presented. We also calculated an estimate of relative effective population size, θ = *N*_*e*_μ, where *N*_*e*_ and *m* are defined as above and μ is the mutation rate per generation at the locus considered.

## Results

### Geological History and Genetic Population Structure

Our data provide mixed support for the hypothesis that *active volcanism fosters genetic diversity*. Populations were found to be highly structured overall, including populations of species found in the *kīpuka* region (Table 2A). Genetic variance among populations, $\Upphi_{ST}$, was significantly greater than 0 at all scales for nearly all species examined, including 3 of 4 species from the *kīpuka* region. For forest specialists, genetic variance among populations in the *kīpuka* region was less than that among volcanoes on the Big Island (Table 2B). However, this comparison is confounded by geographical distance, which is less in the *kīpuka* region than among volcanoes on the Big Island. In the *kīpuka* region, population subdivision was no more pronounced among specialists of forest habitats (L1, L3, L4), than among populations of the habitat generalist (L6, *T. quasimodo*). However, previous analyses of a subset of these species (L3 *T. anuenue,* L4 *T. brevignatha*) found that the amount of genetic differentiation among fragments was greater than that predicted by distance alone in nearby continuous mature wet forest (Vandergast et al*.* 2004). For the 2 species where we had sufficient data for estimates of migration within the *kīpuka* region, *N*_*e*_ *m* among populations was 5.0 for L3 *T. anuenue*, a habitat specialist, and 4.9 for L5 *T. quasimodo*, a habitat generalist, both within the range of estimates of *N*_*e*_ *m* between other populations of these species and others on the Big Island. The relative genetic diversity within populations, θ, ranged from 0.003 to 0.46, though there were no apparent patterns of θ associated with the *kīpuka* region in comparison with other areas.

**Table 2:** Scales of geographical population structure

A. Proportion of variation distributed among populations, $\Upphi_{ST}^{'}$, at different spatial scales^a^					
Species	Marker	Between islands	Within Maui Nui	Within Big Island	Within *kīpuka* region
L1 *Th. grallator*	Allozymes	0.46***	0.37***	0.19***	–
	CO1/16S/ND1	0.69***	0.56***	0.30***	0.05***
L2 *Ariamnes* species	Allozymes	–	–	–	–
	COI	0.37***	0.57***	0.05	–
L3 *T. anuenue & T. kikokiko*	Allozymes	0.36***	–	–	–
	COI	0.98***	–	0.23***	0.041***
L4 *T. brevignatha & T. macracantha*	Allozymes	–	0.46***	–	–
	COI	0.93***	0.76***	0.16*	0.000
L5 *T. quasimodo*	Allozymes	0.06***	0.15***	0.34***	–
	COI	0.74***	–	0.09***	0.037***

B. Comparison of genetic variance among populations, $\Upphi_{ST}^{'}$, at different scales for COI data. Includes among 4 lineages of habitat specialists (L1–L4, excluding L5 *T. quasimodo* and L6 *T. hawaiensis*)		
Species set 1 (mean)	Species set 2 (mean)	Results^b^
1. Within Maui (0.63)	>Within Big Island (0.18)	t = 4.13, *p* < .027*
2. Within Big Island (0.18)	>Among *kīpuka* (0.03)	t = 10.6, *p* < .004**
3. Between islands (0.74)	>Within Big Island (0.18)	t = 3.60, *p* < .035*
4. Between islands (0.74)	>Within Maui (0.63)	t = 0.44, *p* < .35
5. Between islands mtDNA (0.74)	>Between islands nuclear (0.41)	Insufficient sample size

C. *N*_*e*_ *m*, estimated relative gene flow among populations				
Species	Marker	Within Maui Nui	Within Big Island	Within *kīpuka* region
*L1 Th. grallator*	Allozymes	2.6–8.1	3.3–7.1	–
	CO1/16S/ND1	0.6–2.1	1.9–4.2	–
L2 *Ariamnes species*	Allozymes	–	–	–
	COI	–	0.3–5.3	–
L3 *T. anuenue* & *T. kikokiko*	Allozymes	–	–	–
	COI	–	11.7	5.0
L4 *T. brevignatha & T. macracantha*	Allozymes	5.6	–	–
	COI	0.3	0.3–7.0	–
L5 *T. quasimodo*	Allozymes	6.4	6.3–6.5	–
	COI	–	0.3–13	4.9
L6 *T. hawaiensis*	Allozymes	–	–	–
	COI	–	12.0	–

^a^See text for description of analyses and calculations, * *p* < 0.05, *** *p* < 0.001

^b^Arc-sin square-root transformed before analysis, *df* = 2, one-tailed *t* test, * *p* < 0.05, ** *p* < 0.01

Our results provide more convincing evidence for the hypothesis that *volcanic maturation promotes opportunity for isolation*. As predicted, genetic variance among populations on volcanoes within Maui Nui was greater than among volcanoes within the Big Island (Table 2B), consistent with the older age of volcanoes on Maui Nui. However, there were no obvious differences in estimated gene flow, *N*_*e*_ *m*, between populations on different volcanoes within Maui Nui compared to those within the Big Island. The one mid-high elevation lineage on Maui Nui for which we had data, L4 *Tetragnatha brevignatha* and *T. macracantha*, was more highly structured than lower elevation lineages, L1 *Th. grallator* and L2 *Ariamnes species.* We did not have large enough samples of a low elevation species L6 *T. hawaiensis* on Maui Nui to estimate genetic variation among populations. We found increasing isolation with distance from populations within the *kīpuka* region on the Big Island, to populations between volcanoes on both the Big Island and Maui Nui, and to populations between different islands. While genetic structure was greater for mitochondrial COI than for nuclear allozymes, an observation consistent with presumed lower gene flow by females and smaller effective population size, we did not have a sufficient number of comparisons for a statistical test. One species, the habitat generalist L5 *T. quasimodo*, showed a large proportion of genetic variation among populations between islands compared to within islands, as predicted by its generalist habit.

## Discussion

This study highlights the role of dynamic habitats in fostering both diversity and divergence of species. Moreover, the results are consistent with theory that indicates that the relative importance of these effects depends on the spatial and temporal dynamics, here dictated by geological history of the Hawaiian archipelago, and the biology of particular species, including habitat affiliation and patterns of migration.

### Impact of Dynamic Habitats in a Landscape Mosaic: The *kīpuka* Region

In this study of genetic structure of 6 spider lineages we found evidence that dynamic habitats can contribute to population structuring, even with substantial gene flow. Our results from both mitochondrial and nuclear markers show that genetic structure was significant at nearly all scales, including among populations in the *kīpuka* region (Table 2A). For these species, including habitat specialists (L1 *Theridion grallator* and L3 *T. anuenue*) and a habitat generalist (L5 *T. quasimodo*), the results are consistent with the idea that habitat dynamics imposed by volcanic activity contributes to the maintenance of genetic variation.

Indeed, previous work shows that some genetic differentiation can exist even between *kīpuka* separated by <100 m and that differentiation among fragments is greater than that found by distance alone in nearby continuous mature wet forest (Vandergast et al. 2004). However, the isolation provided by these fragments is not likely to translate into species formation for the simple reason that the isolation is short-lived on an evolutionary time scale: The *kīpuka* existing today—which are currently very obvious, surrounded by scrub forest on 150-year old lava flows—will shortly become reconnected. Forest dominated by closed canopy *Metrosideros polymorpha* can develop in 300–400 years on new lava in windward areas of the Big Island (Drake and Mueller-Dombois 1993). At the same time, forests in this area have experienced similar episodes of fragmentation for at least 400,000 years (Lockwood et al. 1988; Carson and Clague, 1995). Therefore, while allopatry caused by lava flows is clearly playing a role in species formation (Carson 1978), that role is likely to be more important in maintaining genetic diversity than in separation of populations.

Our limited estimates of genetic connectivity among populations within the *kīpuka* region suggest that populations in this area remain connected, despite habitat structuring. Estimated levels of migration among *kīpuka* populations were relatively high for the 2 species for which we had sufficient data (L3 *T. anuenue*, *Nm* = 5.0 and L5 *T. quasimodo*, *Nm* = 4.9). These estimates likely reflect ongoing gene flow, but possibly also the relatively small amount of time since geographic isolation. Theoretical work suggests that estimates of gene flow from measurements of genetic divergence are valid only after time sufficient for drift and migration to reach equilibrium, which may be on the order of 4 × *N*_e_ generations, where *N*_e_ = effective population size (Slatkin 1987). While the older volcanoes on the Big Island date back to 0.4MY and older on Maui Nui, populations in the *kīpuka* region are less likely to be at equilibrium.

**Fig. 3:**
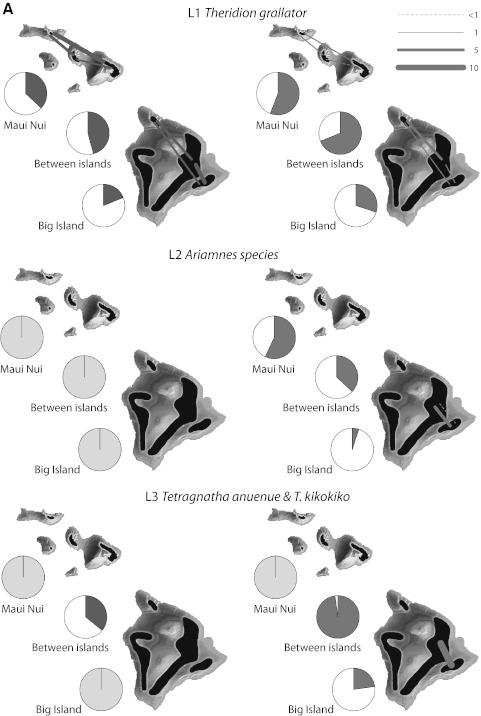

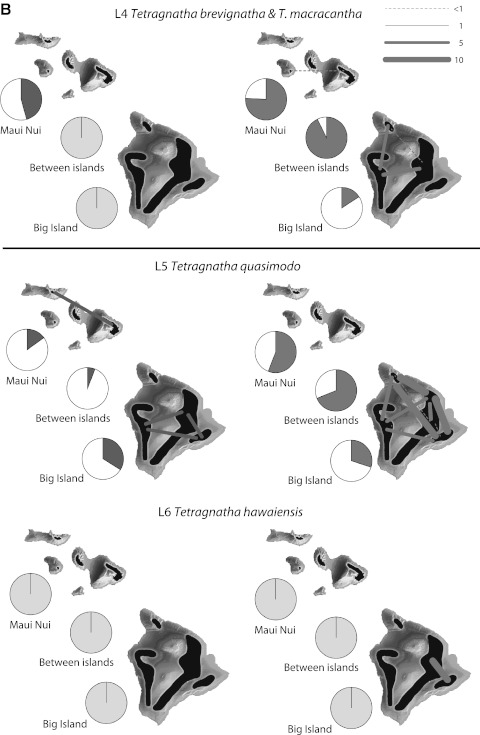
Genetic differentiation at different spatial scales based on data from nuclear allozymes (*red*, *left side*) and mitochondrial COI sequences (*blue*, *right side*). Pies show estimated $\Upphi_{ST}^{'}$, the standardized proportion of genetic variation distributed among populations for volcanoes on Maui Nui, between islands, and among volcanoes on the Big Island; where comparisons were not possible because of limited samples or localities, pies are gray. Thickness of lines on the maps show the magnitude of pairwise gene flow (*N*_*e*_ *m*) between populations estimated from nuclear allozymes (*red*, *left*) and mitochondrial COI sequences (*blue*, *right*), where sample sizes permitted. See text and Fig. 2 for descriptions of each lineage (Color figure online)

Although we do not have detailed population data for L6 *T. hawaiensis*, it is clear that there is considerable movement in this species between populations within islands (Table 2C; Fig. 3), and even between islands (R.G. Gillespie, unpublished data). There are at least two factors that could account for this effect, both as a consequence of the low elevation habitat occupied by the species. First, continuous low elevation forest habitat has been available across the islands of the Maui Nui complex for a long time, presumably also with little isolation between these habitats and similar habitats on the older islands of Oahu and Kauai. As a result, there would have been little impediment to connectivity, except by lava flows. Alternatively, the more heavily impacted lower elevations have caused mixing of previously isolated populations (Shi et al. 2011). Separating the role of these two effects will require more detailed population genetic analyses of populations of *T. hawaiensis.*

### The Importance of the Spatial and Temporal Scale of Separation

This study illustrates the importance of spatial and temporal scale on differentiation among populations. On the Big Island, population structure among volcanoes was greater than among populations within the *kīpuka* area for all 4 species of habitat specialists studied at both scales. These results might be expected given the geographic distances involved (Fig. 1), but also would suggest a temporal framework to colonization in which individuals reach new habitat as it appears, with isolation increasing over time subsequent to colonization of the habitat. On the Big Island, the effect of isolation between volcanoes may have been exacerbated by recent forest clearing (Parker Ranch 1912–1947, James Glover 1947–1958, and Damon Estate 1958–2000), which has resulted in large areas of pasture separating remnant forest (McDaniel et al. 2011).

Populations between volcanoes on Maui Nui showed more genetic structure than those between volcanoes on the Big Island. There are several possible explanations. First, building on the finding of a progression (highest connectivity among the youngest volcanoes), disjunction should increase with island age, as we find. Alternatively, because of its geological history, the lowland areas separating the volcanoes of Maui Nui have always been more expansive than those on the Big Island (Price and Elliott-Fisk 2004), so there has always been greater geographical separation between volcanoes on Maui Nui.

At a larger scale, population divergence for habitat specialists (L1–L4) was greater between the islands of Maui Nui and the Big Island, than among volcanoes within Maui Nui or the Big Island (Table 2B; Fig. 3), consistent with isolation by distance. Within islands, colonization likely occurs quickly as new habitat forms, but the formation of an entirely new island, to which there has never been a historic forested connection, imposes a much greater barrier (Carson and Clague 1995). One interesting exception to this pattern is L5 *T. quasimodo*, a habitat generalist that occurs in wet and dry forest from 700 to 2,300 m elevation. The extensive COI data indicates that populations of this species have high connectivity between volcanoes on the Big Island; the less-well sampled allozyme data from the same sites indicates a much larger separation. In contrast, the better-sampled allozyme data from Maui Nui indicate high connectivity between distant volcanoes as well as between Maui Nui and the Big Island. Insights into this discrepancy come from nuclear intron sequence data (Gillespie et al. 1998), which suggest that each island has been colonized more than once—thus, the Big Island populations of *T. quasimodo* may not be closest genetically to those on Maui itself. This interpretation of a complex population history highlights the critical need for broad sampling of highly mobile species.

### Habitat Dynamics, Speciation and Community Structure

The dynamic nature of volcanic landscapes has consequences for both species differentiation and community assembly (Gruner 2007). As noted above, at the small spatial and temporal scale of the *kīpuka* area, genetic isolation in the absence of diversifying selection is unlikely to result in speciation, given the timeframes of habitat regeneration that occurs on new flows. Populations that expand in the maturing habitat will reconnect and re-establish gene flow in the absence of other isolating factors. However, population growth can increase the relative importance of even weak directional selection (Slatkin 1996; Otto and Whitlock 1997). Further, directional selection may be associated with the novel ecological conditions that organisms encounter under recent habitat fragmentation. For example, previous work found significant variation among Hawaiian *kīpuka* in spider species composition (Vandergast and Gillespie 2004), prevalence of parasitism (Vandergast and Roderick 2003), and diversity and density of arthropods (D Gruner, personal communication). The loss of potential competitors and changes in trophic interactions may allow remaining species to exploit and adapt to previously occupied niches. Additional opportunities for adaptation exist at the boundaries of new lava flows, where closed forest and open lava habitats abut.

Less well studied in the context of these dynamic landscapes and associated adaptations is the nature of ecological community assembly. For example, models used to reproduce food web structures have long focused on equilibrium conditions without incorporating dynamical or adaptive components (Williams and Martinez 2000; Cattin et al. 2004). Because of the complexity of the problem, adaptation has not yet been incorporated into food web models with multiple species. Most often, models that tackle food web structures do not include dynamical aspects, interaction strengths or population densities (Cohen 1989; Williams and Martinez 2000; Cattin et al. 2004). These models are usually parameterized using community scale characteristics such as species diversity and connectance to match other community scale descriptors, so that making a link with individual scale processes such as adaptive foraging or even population dynamics is not straightforward.

Loeuille (2010) has examined conditions under which adaptation is relevant to food web structure, and shown that in such situations, it produces additional indirect demographic and adaptive effects that both increase structuring effects and decrease their predictability. A pioneering study that incorporated evolutionary change into analyses of food webs simulated food web assembly through speciation and the evolution of traits (body size and niche width) that determine ecological interactions (Ingram et al. 2009). This study showed that conditions favoring a generalist strategy also make the food web more invasible to new species, leading to constant high rates of species turnover. Ingram et al. (2009) suggested that the higher variability and species turnover in food webs with generalist/omnivorous species may be because of a flattened adaptive landscape, which increases the proportion of trait space that can maintain viable populations. This relationship may depend on variation in environmental features such as structural complexity of the environment (Ritchie and Olff 1999). As a consequence, if environmental heterogeneity allows size-based partitioning of habitat, consumers may be penalized for a generalist foraging mode because of the costs of maintaining foraging tactics at multiple spatial scales; such a community would have relatively low levels of omnivory and high temporal stability. Clearly, founder events and fragmentation can induce major ecological changes, and future studies must incorporate how associated changes in diversity and species interactions are transmitted into selection and adaptive shifts, and at what time scale, and how they interact with changes in genetic and population structure.

## Conclusions

The results shown here are consistent with the hypotheses that (1) active volcanism maintains and fosters genetic diversity, although through a complicated process involving population subdivision in the context of migration and admixture; and (2) volcanic maturation results in greater isolation of populations over time, thus promoting and increasing opportunities for isolation. Future studies must focus explicitly at the population level to gain more precise insights into the temporal framework of connectivity and isolation under which differentiation can occur (see Vuilleumier et al. 2010), and to rule out possible effects of recent human influences in affecting this pattern (Gillespie et al. 2008). In addition, it will be important to integrate ecological shifts associated with the dynamic landscape, and determine how they interact with population genetic modifications to shape species adaptation and differentiation.
